# Impacts of Kv3.1 Channels on Action Potentials in Fusiform Neurons From the Dorsal Cochlear Nucleus of Mice Before and After Hearing Onset

**DOI:** 10.1007/s10162-026-01067-7

**Published:** 2026-08-12

**Authors:** Mario Henrique Lopes Duarte de Oliveira, Alan Eidi Sasaki, Leonardo Calaça Arruda Vanderlei, Ricardo Mauricio Leão

**Affiliations:** Laboratory of Neurophysiology and Synapse, Department of Physiology, School of Medicine of Ribeirão Preto, University of São Paulo, Ribeirão Preto, SP Brazil

**Keywords:** Kv3, Action potential, Cochlear nucleus, Hearing, Development

## Abstract

**Purpose:**

Kv3.1 channels are high-voltage-activated potassium channels that facilitate high-frequency firing in auditory brainstem neurons. Fusiform neurons in the dorsal cochlear nucleus (DCN) can fire rapid trains of action potentials and express Kv3.1 channels. Their ability to fire high-frequency action potential trains increases after hearing onset at postnatal day 14. Simultaneously, the action potentials become shorter and faster. We tested whether Kv3.1 channels drive the developmental maturation of action potentials in DCN fusiform neurons.

**Methods:**

We used Swiss female mice from postnatal day 8 to 25 and divided them into pre-hearing (< P14) and post-hearing (> P14). We measured Kv currents and action potentials in whole-cell patch-clamp. Tetraethylammonium (TEA) was applied at a concentration of 1–5 mM to block Kv3 channels, and we detected the expression of Kv3.1b channel subunits by immunocytochemistry.

**Results:**

We found increased Kv3.1b subunit expression in the DCN after hearing onset, suggesting that this subunit could be responsible for the electrophysiological changes in post-hearing fusiform neurons. TEA-sensitive high-threshold currents were a significant component of voltage-dependent potassium currents in both pre- and post-hearing fusiform neurons, with similar magnitudes in both groups. However, blocking Kv3 currents with 1–5 mM TEA broadens action potential waveforms in neurons from both groups while maintaining the developmental differences between groups. On the other hand, TEA was more effective at reducing the firing of post-hearing neurons.

**Conclusions:**

We conclude that the rise in Kv3.1b channel expression in DCN fusiform neurons after hearing does not account for the shorter, faster action potentials observed at that time, but could contribute to their ability to fire action potentials at high frequencies.

## Introduction

The dorsal cochlear nucleus (DCN) integrates excitatory signals from auditory, somatosensory, proprioceptive, and vestibular sources. The neurons in this nucleus respond to spectral cues, which are variations in the sound spectrum caused by the interaction of sound with the external ear. They combine these cues with sensory inputs to locate the sound source, primarily in the vertical plane [1]. The fusiform neurons are the principal projection neurons of the DCN. It receives inputs from both the auditory nerve and the multimodal pathway via the parallel fibers, integrating these inputs to support the computational tasks performed by the DCN. Fusiform neurons fire action potentials steadily in response to current injection, showing no accommodation or depolarization block of firing [2–4]. The DCN fusiform neurons undergo a series of electrophysiological refinements of their action potentials after the opening of the ear canal and the start of hearing at postnatal day 14 (P14), becoming faster and shorter. Additionally, there is an increase in these neurons' ability to fire action potentials at high frequencies after P14 [4].

Kv3 subunits of voltage-gated potassium channels are expressed in fast-spiking neurons, enabling these neurons to sustain regular, high-frequency action potential firing [5–12]. These channels are highly expressed in auditory brainstem nuclei, with a prominent expression of the Kv3.1 subunit [13–15]. The expression of these channels changes with the development of the central nervous system [16, 17]. The Kv3 family of voltage-dependent potassium channels comprises four subunits (Kv3.1–4), characterized by activation at very high membrane potentials, rapid activation and deactivation, and slow inactivation [18]. This allows neurons expressing these channels to have short refractory periods, enabling them to fire action potentials at high frequencies [7]. Kv3.1 channels undergo alternative splicing, producing two isoforms, Kv3.1a and Kv3.1b, that differ in C-terminus length [19]. Consequently, Kv3.1b is longer and contains a PKC phosphorylation site. The Kv3.1b isoforms interact with Ankyrin G and are primarily localized in the axonal initial segment and nodes near voltage-dependent sodium channels, regulating action potential firing. In contrast, Kv3.1a neurons are located in the somato-dendritic membrane [20, 21].

The DCN is a central nucleus for tinnitus generation[22, 23], and an increased spontaneous firing frequency of DCN neurons is seen in animal models of tinnitus [24, 25]. Kv3 currents are crucial for regular firing in DCN fusiform neurons [26]. In rodents exposed to sound trauma, which can induce tinnitus, fusiform neurons present less ability to fire at high frequencies and express a greater tendency for burst-firing by a reduction of Kv3 currents[27]. In the inferior colliculus, the application of a modulator of Kv3 currents reduces the increased spontaneous firing following noise exposure [28]

Considering the importance of Kv3 channels for high-frequency firing of DCN fusiform neurons, and the developmental changes in the DCN fusiform neurons we observed during the critical period for hearing and tinnitus development [29, 30] we, herein, investigated whether the developmental changes in the electrophysiological properties of these neurons in mice are caused by increased Kv3 channel currents following hearing onset.

## Methodology

### Animals

Pregnant Swiss female mice from the University of São Paulo's central animal facility in Ribeirão Preto were housed and monitored daily for the birth of their offspring. When the litter size exceeded eight pups, the remaining pups were euthanized to minimize developmental variability. The animals were kept in the FMRP-USP mouse maintenance facility, where they were maintained on a 12/12-h light/dark cycle with food and water provided ad libitum. Mice aged post-natal days 8 to 25 (P8-25) were used in the study. The ARRIVE guidelines were followed in the design and analysis of the experiment. All animal procedures were approved by the Ethics Committee on Animal Experimentation (CEUA) of the School of Medicine of Ribeirão Preto (CEUA, Protocol 1289/2024R1).

### Slice Preparation

Mice were sacrificed by decapitation after deep anesthesia with isoflurane. The brainstems were removed after cutting the vestibulocochlear nerve. The brainstems were submerged in melted agarose (2.5% in aCSF) at 38 °C, and the temperature was rapidly lowered using a metal block to solidify the agarose. The block was mounted in a compresstome (VF300-0Z, Precisionary Instruments, USA), and 200 µm thick coronal brain slices containing the dorsal cochlear nucleus, DCN, were cut in an ice-cold solution containing (in mM) 87 NaCl, 2.5 KCl, 1.25 NaH_2_PO_4_, 25 NaHCO_3_, 0.2 CaCl_2_, 7 MgCl_2_, 25 glucose, and 75 sucrose, pH 7.4 when oxygenated with 95% O_2_/5% CO_2_. Slices were incubated in artificial cerebrospinal fluid (aCSF) for 30 min at 35 °C and then at room temperature (25 °C). The aCSF contained (in mM) 120 NaCl, 2.5 KCl, 1.25 NaH_2_PO_4_, 25 NaHCO_3_, 2 CaCl_2_, 1 MgCl_2_, and 10 glucose, with a pH of 7.4 when oxygenated with 95% O_2_/5% CO_2_ (osmolality, 310 mOsmol/kg H_2_O).

### Electrophysiology

Slices were positioned on the stage of an upright microscope (BX51W, Olympus, Japan), and neurons were visualized under oblique illumination with a 63 × water immersion objective. Slices were continually perfused (~ 1 mL/min) with normal aCSF. Recordings were obtained with borosilicate microelectrodes (BF150-86–10, Sutter Instrument Company, USA) with resistance between 2 and 4 MΩ when filled with internal solution (in mM) 130 K-gluconate, 20 KCl, 10 Na_2_-phosphocreatine, 10 HEPES, 0.1 EGTA, 2 Mg-ATP, and 0.2 Na-GTP, pH 7.4 adjusted with KOH, with a final osmolality of 295 mOsmol/kg H_2_O. Fusiform neurons were identified by their location in the principal layer of the DCN and by their biophysical characteristics [31, 32]. Whole-cell patch-clamp recordings were performed using an Axopatch 200B (Molecular Devices) connected to a Digidata 1440 A board (Molecular Devices). Whole-cell recordings started at least 5 min after attaining whole-cell configurations. Series resistance and cell capacitance were measured using responses to 5 mV depolarizing voltage steps from a holding potential of −75 mV. Whole-cell capacitance was canceled, and series resistance was checked regularly. Series resistance was compensated in the range of 50 to 70%, and the maximum uncompensated series resistance allowed was 20 MΩ. All recordings were low-pass filtered (3 kHz, Bessel) and acquired at a sampling rate of 50 kHz using Clampex software (version 11, Molecular Devices).

Membrane voltage recordings were performed in the fast I-Clamp mode. Active neurons are neurons that present regular firing without the injection of depolarizing current. Quiet neurons were neurons with a stable resting membrane potential, with no firing or episodic firing at rates less than 1 Hz [33]. We did not differentiate these groups in the analysis.

Current-clamp recordings were performed at a controlled temperature of 35- 36 °C using an inline heating system (Warner Instruments). Voltage-current (IV) relationships were obtained by injecting 1.5 s current pulses with 25pA steps from −100pA. Quiet neurons were maintained at their resting membrane potential (RMP). For active neurons, DC current (−100 pA to −300 pA) was injected to hyperpolarize the membrane (to approximately −70 mV) and to stop action potentials from firing.

Voltage-clamp recordings were performed at room temperature (22–25 °C). Current–voltage (IV) relationships were recorded, maintaining the neurons at a holding potential of −70 mV, and delivering 1500 ms depolarizations from −60 to 20 mV in 10 mV steps every 10 s.

### Drugs

Drugs were gravity-driven applied at the rate of 1 mL/minute. TEA and Cadmium Chloride were obtained from Sigma, and tetrodotoxin (TTX) from Alomone Labs. Stock solutions (1000x) were diluted into aCSF to achieve the desired final concentrations.

### Immunofluorescence

Four P8 mice, representing the pre-hearing group, and four P20 mice, representing the post-hearing group, were used. Animals were deeply anesthetized with ketamine (200 mg/kg) and xylazine (10 mg/kg) by intraperitoneal injection. Once sedation was confirmed, they underwent transcardiac perfusion with cold physiological saline, followed by 4% paraformaldehyde (PFA) in saline. After perfusion, mice were decapitated, and the brainstems were extracted and immersed in 4% PFA for 60 min. They were then stored in a 30% sucrose solution until completely submerged (in 24–48 h). Next, the brainstems were immersed in isopentane frozen with dry ice for 30 s and stored in a freezer at −20 °C. The brainstems were sectioned into 35 µm slices using a cryostat at −20 °C. These slices were stored in tubes containing 1.5 mL of antifreeze solution (30% sucrose, 0.2 M PB, and ethylene glycol) at −20 °C until used for immunostaining.

For antibody incubation, the slices were first washed in permeabilization solution (PBS + 0.3% Triton X-100) and then incubated for 1 h in blocking buffer (0.3% (v/v) Triton X-100 and 0.4% (v/v) normal goat serum in PBS) at room temperature with gentle agitation (75 rpm). Subsequently, the slices were incubated overnight at 4 °C in free-floating conditions with anti-Kv3.1b polyclonal antibodies (1:1500, rabbit, Alomone Labs, APC-014) or PhosKv3.1b (1:1500, rabbit, R&D Systems, PPS016) diluted in blocking buffer. After incubation, the slices were washed with PBS and then incubated for 2 h at room temperature in blocking solution containing secondary goat anti-rabbit IgG-Alexa Fluor 594 antibodies (1:3000, Vector Laboratories), with agitation and protection from light. After incubation, the slices were washed with PBS and mounted on slides. The slides were coverslipped with VectaShield containing DAPI (Vector Laboratories), and images were acquired using a fluorescence microscope (Leica DM 4500B) at 10 × magnification and a Leica Stellaris 5 confocal microscope at 63 × magnification with a 5 × digital zoom. One image (1024 × 1024, xy pixel size 0.036 µm × 0.036 µm) was acquired for each layer per DCN.

### Data Analysis

Electrophysiological data were subtracted offline for a liquid junction potential of 10 mV, which was determined experimentally as in [34]. Action potentials were analyzed in Clampfit as in [35]. The first action potential fired at rheobase was used for the analysis. Rheobase was defined as the minimum current required to trigger an action potential from the resting potential (quiet neurons) or from a potential near −70 mV in active neurons. The action potential threshold was defined as the membrane potential at which dVm/dt reached 10 V/s during the AP upstroke. Action potential amplitude was the difference between the threshold and the action potential peak. The maximum rise slope was the largest positive slope from the baseline to the peak. The maximum decay slope was the largest negative slope between the AP peak and the afterhyperpolarization peak. Decay time was the difference between the AP peak and the afterhyperpolarization peak. Half-width was the duration of the action potential at half of its amplitude, and the latency was the time between the start of the stimulus and the point at which Vm reached the threshold of the first action potential at rheobase.

Kv currents were analyzed at the last 100 ms. The effect of TEA was quantified after 5 min of perfusion when the drug´s effect stabilized. Cells with visible signs of current run-down were not analyzed. Currents were divided by the cell´s capacitance, determined by the cancellation of the capacitive transients by the amplifier. Current density was expressed as current by capacitance (pA/pF).

Immunofluorescence was analyzed using FIJI/ImageJ software (http://rsb.info.nih.gov). Further, to ensure anatomical homogeneity, 4 to 6 DCN sections per animal were examined for fluorescence intensity. The images were converted to grayscale (8-bit), and the DCN layers (region of interest, ROI) were delineated in each image based on the DAPI staining. Pixel size was calibrated (0.51 µm; resolution: 1040 × 1392), and fluorescence intensity was quantified using integrated optical density. Data were averaged to obtain the mean fluorescence intensity per animal, and values from individual slices were normalized to the mean fluorescence intensity of P8 animals. Fluorescence intensity was expressed as fold change. Non-specific labeling was assessed by incubations with the secondary antibody only.

To estimate the total number of Kv3.1b puncta, we used the machine-learning–based image analysis software Ilastik [36]. Grayscale (8-bit) images with brightness and contrast adjustments to enhance the visualization of puncta in each layer were imported from ImageJ into Ilastik, and the “Density Counting” workflow was selected. The parameter Sigma was set to 2.5. Training labels were assigned to foreground (puncta) and background regions, and the results were visualized in real time using the “Live Update” feature until the number of elements was accurately estimated. The box interaction mode was used to obtain predicted object counts and validate their accuracy. The total puncta count for each image was normalized to the mean value of the P8 group. Non-specific labeling was assessed by incubations with the secondary antibody only.

Statistics were performed in GraphPad Prism 10.0. Means were compared with paired or unpaired t-tests. Non-parametric tests (Mann–Whitney or Wilcoxon matched-pairs signed rank) were applied to parameters that did not pass the Shapiro–Wilk normality test, but no significant differences were observed compared with the parametric tests. Proportions were compared using Fisher´s exact test. Outliers were detected with the ROUT test (Q = 1%). The significance level was set at p = 0.05. Data is presented as mean ± SEM.

## Results

### K3.1b Channel Expression Increases After Hearing Onset

**Fig. 1:**
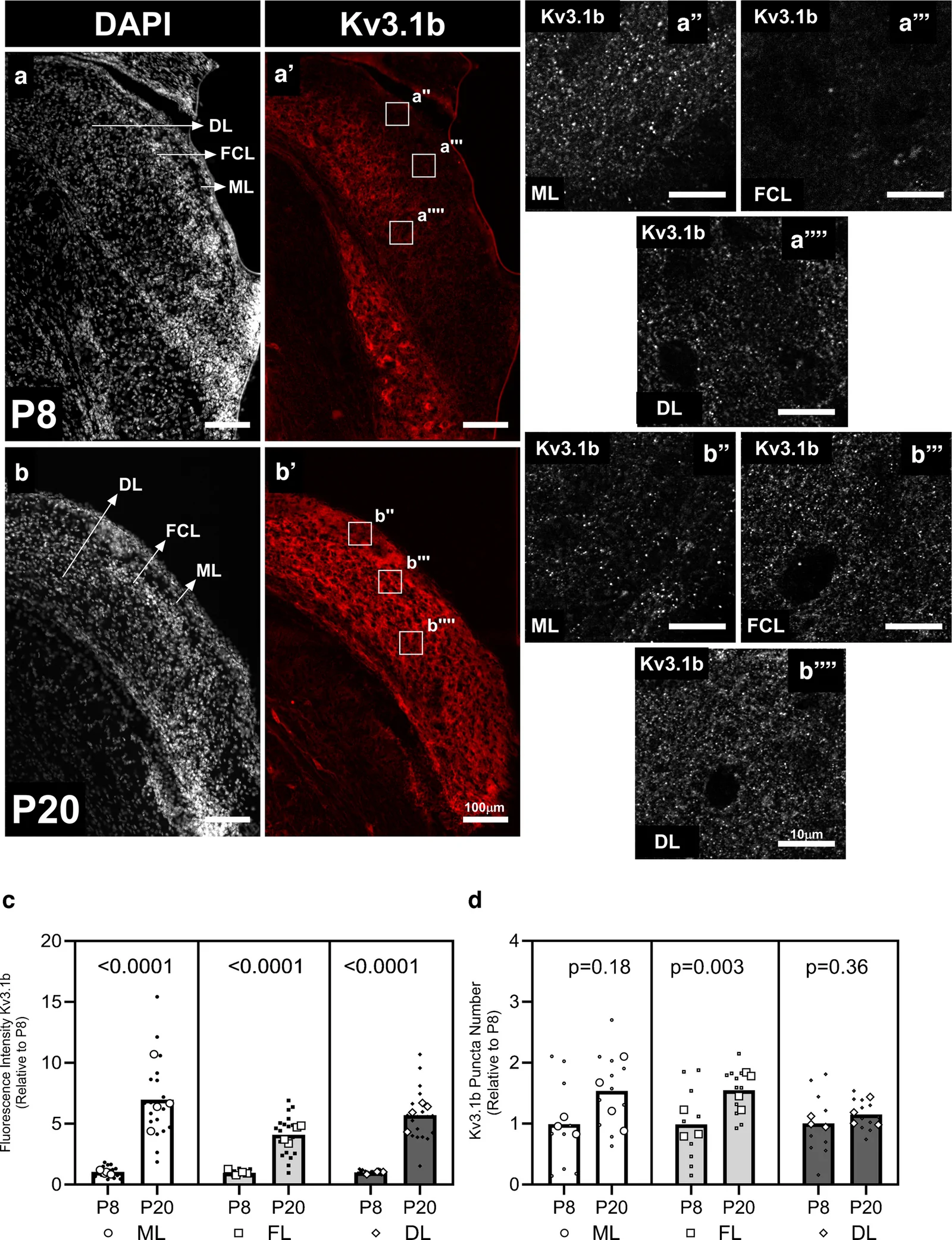
Expression of Kv3.1b channels in the DCN. A. Representative labeling of a DCN of a pre-hearing mouse (**a**) and post-hearing mice (**b**). a,b. DAPI labeling with the 3 layers identified. **a´**,**b´**. Kv3.1b labeling from pre-hearing and post-hearing mouse, respectively. **a´´**, **b´´** Kv3.1b puncta from the molecular layer of a pre-hearing and post-hearing mouse, respectively; **a´´´**, **b´´´** Kv3.1b puncta from the fusiform layer of a pre-hearing and post-hearing mouse, respectively; **a´´´´**, **b´´´´** Kv3.1b puncta from the deep layer of a pre-hearing and post-hearing mouse, respectively. **c**. Quantification of the Kv3.1b immunofluorescence intensity in the molecular, fusiform, and deep layer of the DCN (ML, FL, and DL), big symbols represent the labeling from each animal, and the small points represent the fluorescence from individual DCN sections. **d**. Quantification of Kv3.1b puncta in the molecular, fusiform, and deep layer of the DCN (ML, FL, and DL), big symbols represent the labeling from each animal, and the small points represent the labeling from individual DCN sections

Kv3.1b subunit has been linked to the ability of neurons of the medial nucleus of the trapezoid body (MNTB) to fire at high frequencies [6, 37]. We evaluated the expression of Kv3.1b channel subunit in the DCN by immunocytochemistry. We labeled slices from 4 P8 and 4 P20 animals and evaluated the Kv3.1b expression in the 3 layers of the DCN. We found a substantial increase in Kv3.1b labeling intensity in P20 animals in all 3 layers of the DCN (Fig. 1a-c). We then quantified the number of Kv3.1b-positive puncta using confocal microscopy (Fig. 1 a’ ^–^ ’’’,b’ ^–^ ’’’) and found an increase in the specific number of Kv3.1b puncta specifically in the fusiform cell layer (Fig. 1d).

**Fig. 2:**
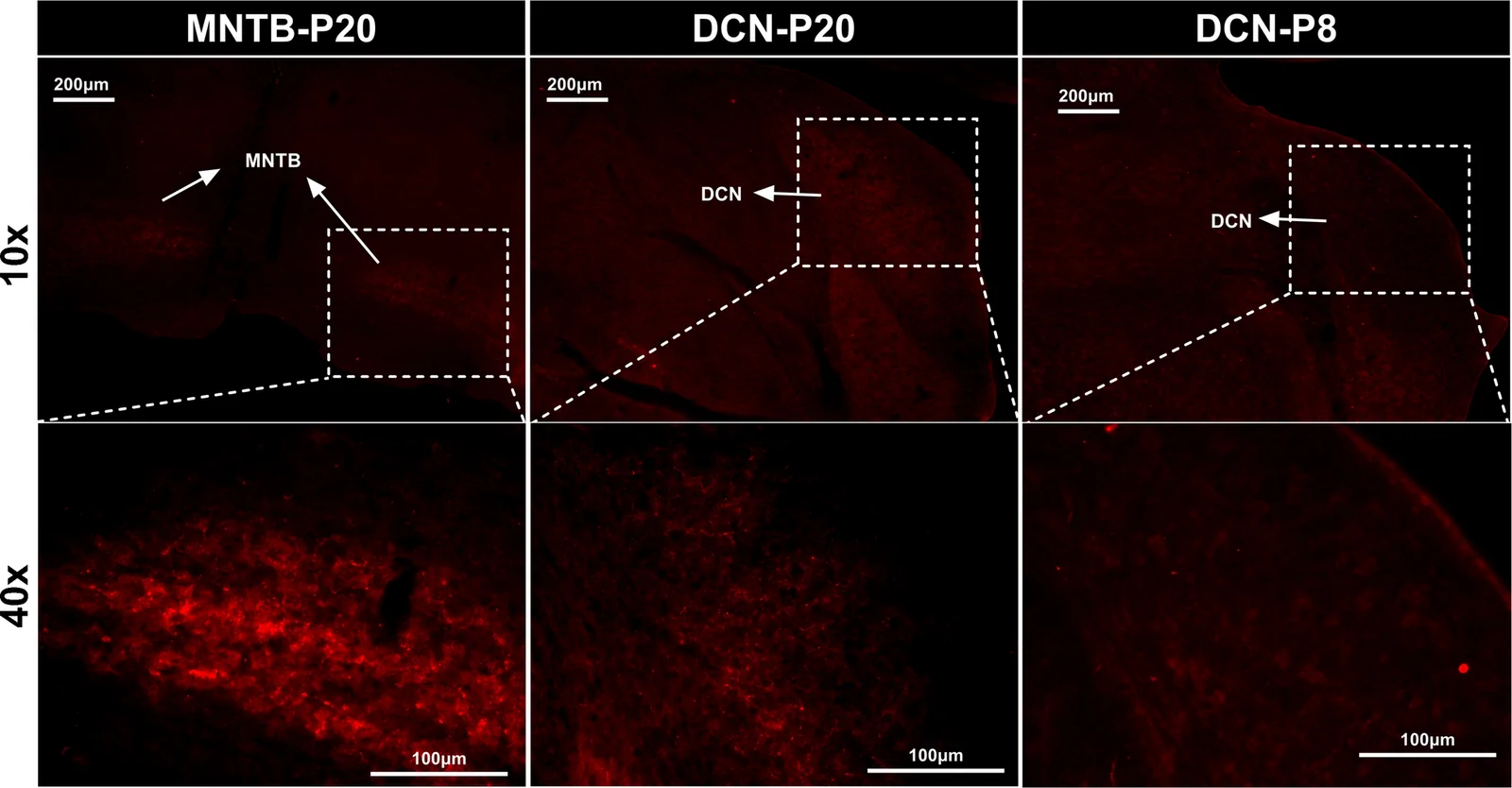
Representative Immunolabelings of phosphorylated Kv3.1b in the MNTB, and DCN from P20 (MNTB and DCN) andP8 (DCN) animals under a 10 × and 40 × objectives

Phosphorylation of Kv3.1b decreases the magnitude of the current, resulting in a better temporal fidelity of medium frequency trains of action potentials but not to high frequency trains [37]. Additionally, exposure to a noisy environment decreases the phosphorylation state of Kv3.1b in MNTB neurons [38]. We then tested the hypothesis that post-hearing DCN neurons are less phosphorylated than pre-hearing neurons using an antibody against the phosphorylated isoform of Kv3.1b neurons. However, we found no significant expression of the phosphorylated form of Kv3.1b channel in the DCN of both age groups (3 animals per group; Fig. 2). To test the efficacy of the antibody, we labeled sections of the MNTB with our antibody and detected the expression of the phosphorylated form of the Kv3.1b channel (Fig. 2). We conclude that Kv3.1b channels are not phosphorylated in the DCN of either pre-hearing or post-hearing mice, at least in our conditions.

### Post-hearing Neurons Express a More Robust Kv Current but with Similar Levels of the TEA-sensitive Component

We measured K_v_ currents after application of TTX (0.5 µM) and CdCl_2_ (100 µM) to block Na^+^ and Na^+^-dependent K^+^ currents, Ca^2+^ and Ca^2+^-dependent potassium currents, respectively. We performed these recordings at room temperature to reduce current oscillations throughout the whole-cell recording. We used 5 mM tetraethylammonium (TEA) to inhibit Kv3 currents.

**Fig. 3:**
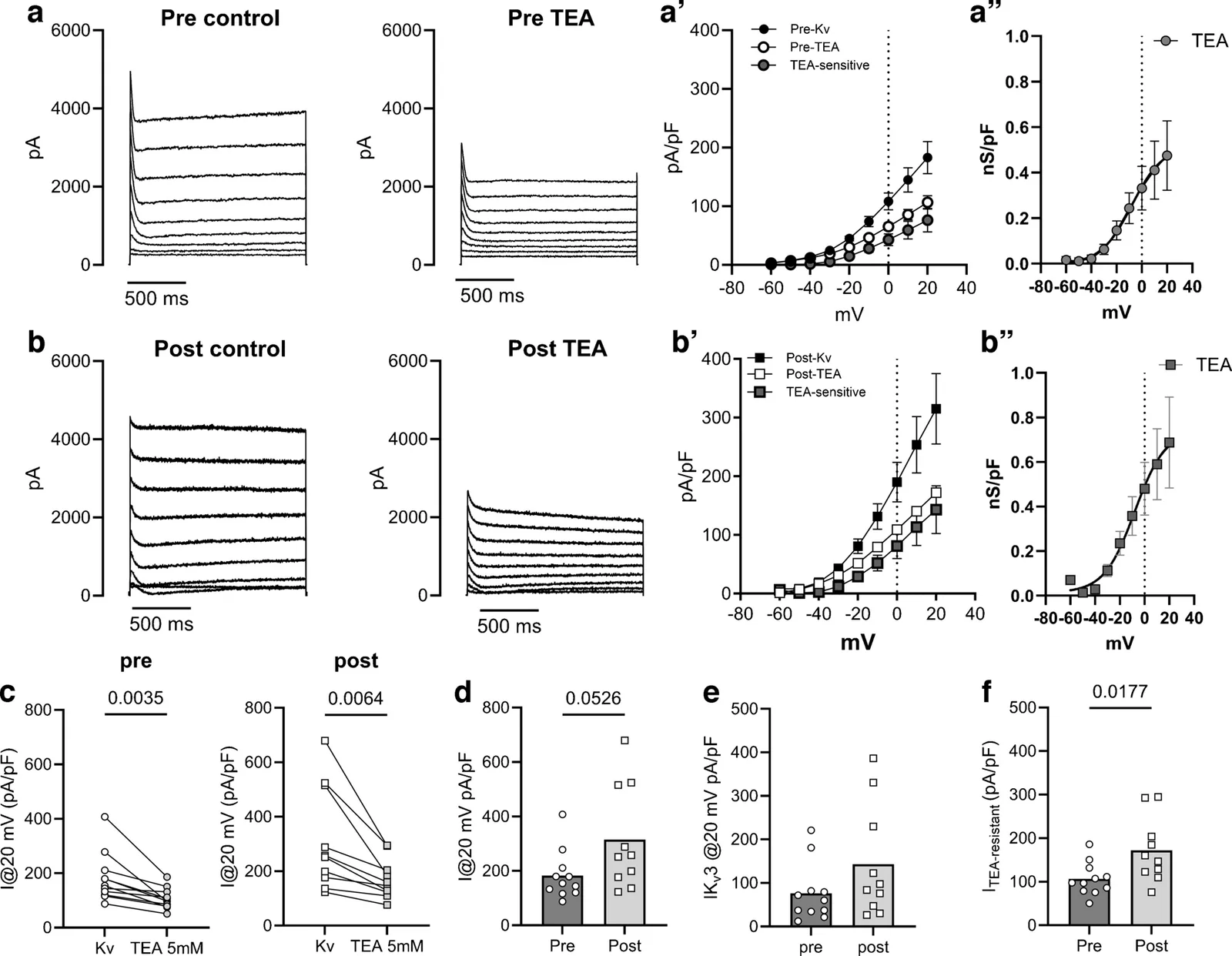
Inhibition of the Kv currents by TEA. **a**. Representative example of a Kv current from a pre-hearing fusiform neuron; **a’** IV relationships of the Kv current before and after application of TEA, and of the TEA-sensitive current of a pre-hearing fusiform neuron. **a´´** Conductance-voltage relationship of the TEA-sensitive current fitted with a Boltzmann function. **b**. Representative example of a Kv current from a post-hearing fusiform neuron; **b’** IV relationships of the Kv current before and after application of TEA, and of the TEA-sensitive current of a post-hearing fusiform neuron. **b’’** Conductance-voltage relationship of the TEA-sensitive current fitted with a Boltzmann function. **c**. individual values of the Kv current elicited by a 20 mV pulse, before and after 5 mM TEA, in pre-hearing and post-hearing fusiform neurons. **d**. Comparison of the Kv current elicited by a 20 mV pulse in pre- and post-hearing fusiform neurons. **e**. Comparison of the Kv3 current (TEA-sensitive) elicited by a 20 mV pulse in pre- and post-hearing fusiform neurons. **f**. Comparison of the TEA-resistant Kv current elicited by a 20 mV pulse in pre- and post-hearing fusiform neurons

The IV relationships of the total K_v_ currents before and after application of TEA 5 mM can be seen in Fig. 3. Total K_v_ currents were bigger in post-hearing neurons (I@20 mV pre-hearing: 182.9 ± 27 pA/pF; post-hearing: 315.3 ± 60 pA/pF. t = 2.07, df = 19, *p* = 0.05, unpaired t-test. n = 11 and 10 respectively; Fig. 3a,b,d). Application of TEA 5 mM significantly inhibited the Kv currents in pre-hearing neurons (t = 3.80, df = 10, *p* = 0.0035, paired t-test, *n* = 11; Fig. 3a,c) and post-hearing neurons (t = 3.5, df = 9, *p* = 0.0064, paired t-test, n = 10; Fig. 3b,c). The TEA-sensitive Kv3 current was not significantly different between the two age groups (I@20 mV pre-hearing: 76.2 ± 20 pA/pF; post-hearing: 143.1 ± 44 pA/pF. t = 1.5, df = 19, *p* = 0.14, unpaired t-test. *n* = 10 and 11 respectively; Fig. 3e). The TEA-sensitive currents had V_50_ values more characteristic of HVA currents in both pre-hearing (−7.89 mV) and post-hearing (−7.97 mV; Figs. 3a’’ and b’’). However, the TEA-resistant current was significantly bigger in post-hearing neurons (I@20 mV pre-hearing: 106.6 ± 11 pA/pF; post-hearing: 172.2 ± 23 pA/pF. t = 2.6, df = 19, *p* = 0.017, unpaired t-test. *n* = 10 and 11 respectively Fig. 3f).

### Kv3 Channels Inhibition Affects the Action Potential Properties Equally in Both Pre- and Post-hearing Neurons, but Does not Alter the Developmental Differences of Action Potentials

We then asked whether Kv3 channels are responsible for the developmental differences observed in the action potential waveforms in fusiform neurons. In Table 1, we present the developmental differences in action potential parameters of fusiform neurons from pre- and post-hearing animals used in these experiments. We found significant differences in rheobase, threshold, decay time, maximum rise slope, maximum decay slope, half-width, and latency, consistent with our previous report [4], but not in AP amplitude.

**Table 1:** Comparison of action potential parameters from fusiform neurons from pre-hearing and post-hearing neurons

	Pre-hearing	Post-hearing
Rheobase (pA)	211 ± 47 (*n* = 9)	75 ± 17 *p* = 0.016 (*n* = 9)
Peak amplitude (mV)	67 ± 5 (*n* = 9)	64 ± 7 *p* = 0.4 (*n* = 9)
Threshold (mV)	−36 ± 1 (*n* = 9)	−40.7 ± 1 *p* = 0.03 (*n* = 8)
Decay time (ms)	0.3 ± 0.03 (*n* = 9)	0.1 ± 0.05 *p* = 0.001 (*n* = 8)
Max. rise slope (mV.ms^−1^)	293 ± 17 (*n* = 9)	365 ± 15 *p* = 0.006 (*n* = 9)
Max. decay slope (mV.ms^−1^)	−193 ± 14 (*n* = 9)	−323 ± 15 *p* = 0.0003 (*n* = 9)
Half-width (ms)	0.44 ± 0.03 (*n* = 9)	0.27 ± 0.0075 *p* = 0.0001 (*n* = 8)
Latency (s)	0.46 ± 0.1 (*n* = 9)	0.07 ± 0.0076 *p* = 0.004 (*n* = 9)

**Fig. 4:**
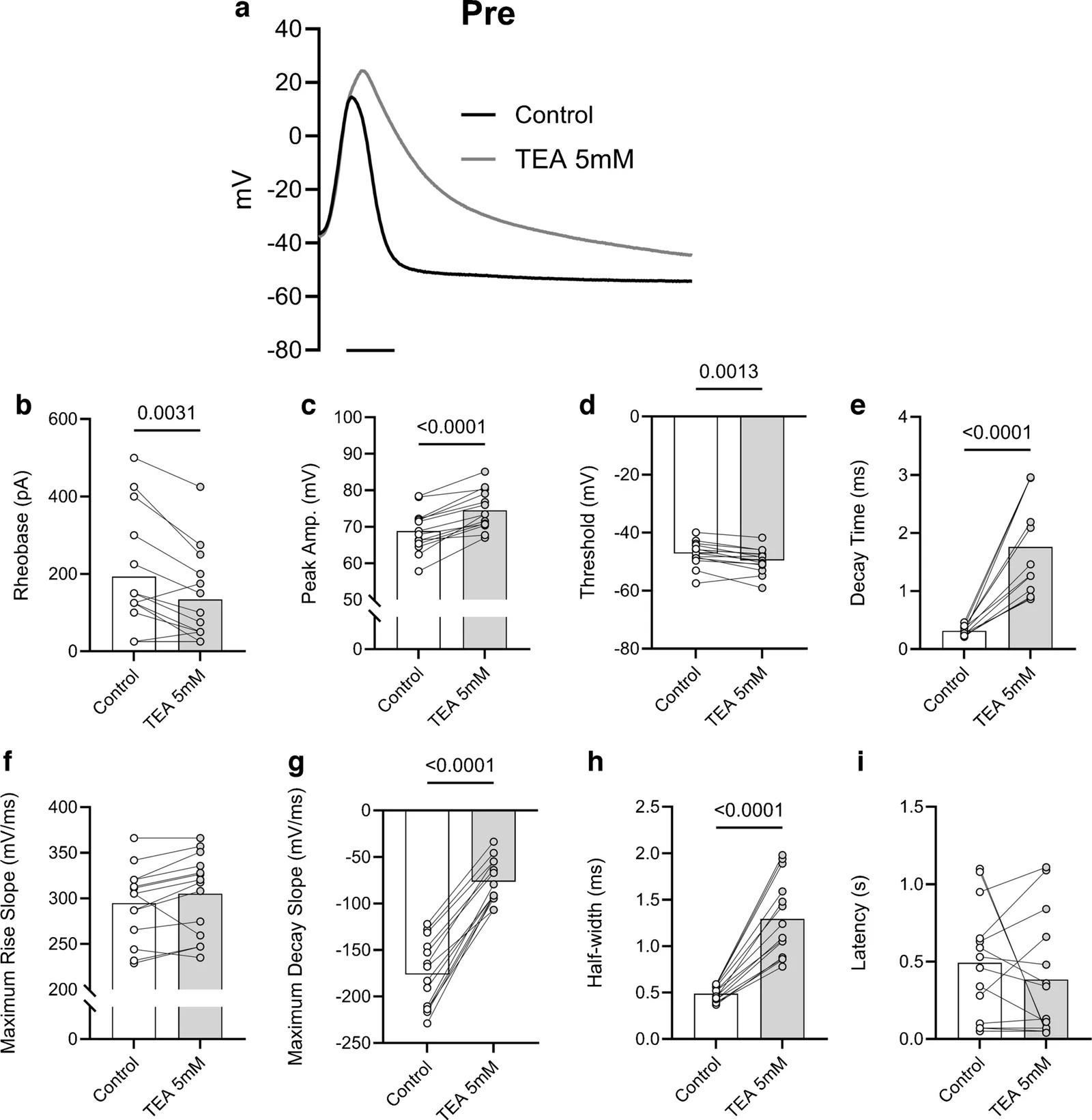
Effect of TEA on action potential parameters of pre-hearing fusiform neurons, **a**. Representative example of a pre-hearing action potential before and after TEA 5 mM; Time scale bar = 1 ms. Effect of TEA 5 mM on: **b**. Rheobase; **c**. Peak amplitude; **d**. Threshold; **e**. Decay time; **f**. Maximum rise slope. **g**. Maximum decay slope; **h**. Half-width; **i**. Latency

Application of TEA 5 mM in pre-hearing fusiform neurons changed various AP parameters (Fig. 4a). TEA decreased the rheobase (from 193 ± 42 pA to 134 ± 32 pA; t = 3.619, df = 13, *p* = 0.03, *n* = 14; Fig. 4b), increased peak amplitude (from 69 ± 1.5 mV to 74 ± 1.4 mV; t = 7.783, df = 13, *p* < 0.0001, *n* = 14; Fig. 4c), decreased threshold (from −47.2 mV to −49.6 mV; t = 4.096, df = 13, *p* = 0.0013; *n* = 14 Fig. 4d), increased decay time (0.3 ± 0.2 ms to 1.8 ± 0.2 ms; t = 6.744, df = 12, *p* < 0.0001; *n* = 13 Fig. 4e), decreased maximum decay slope (from −176 ± 10 mV/ms to −76 ± 6 mV/ms; t = 16.56, df = 13, *p* < 0.0001, *n* = 14; Fig. 4g) and increased half-with (from 0.49 ± 0.2 ms to 1.3 ± 0.1 ms; t = 8,433, df = 13, *p* < 0.0001, *n* = 14; Fig. 4h). Maximum rise slope (Fig. 4f) and latency of the first AP were not affected (Fig. 4i).

**Fig. 5:**
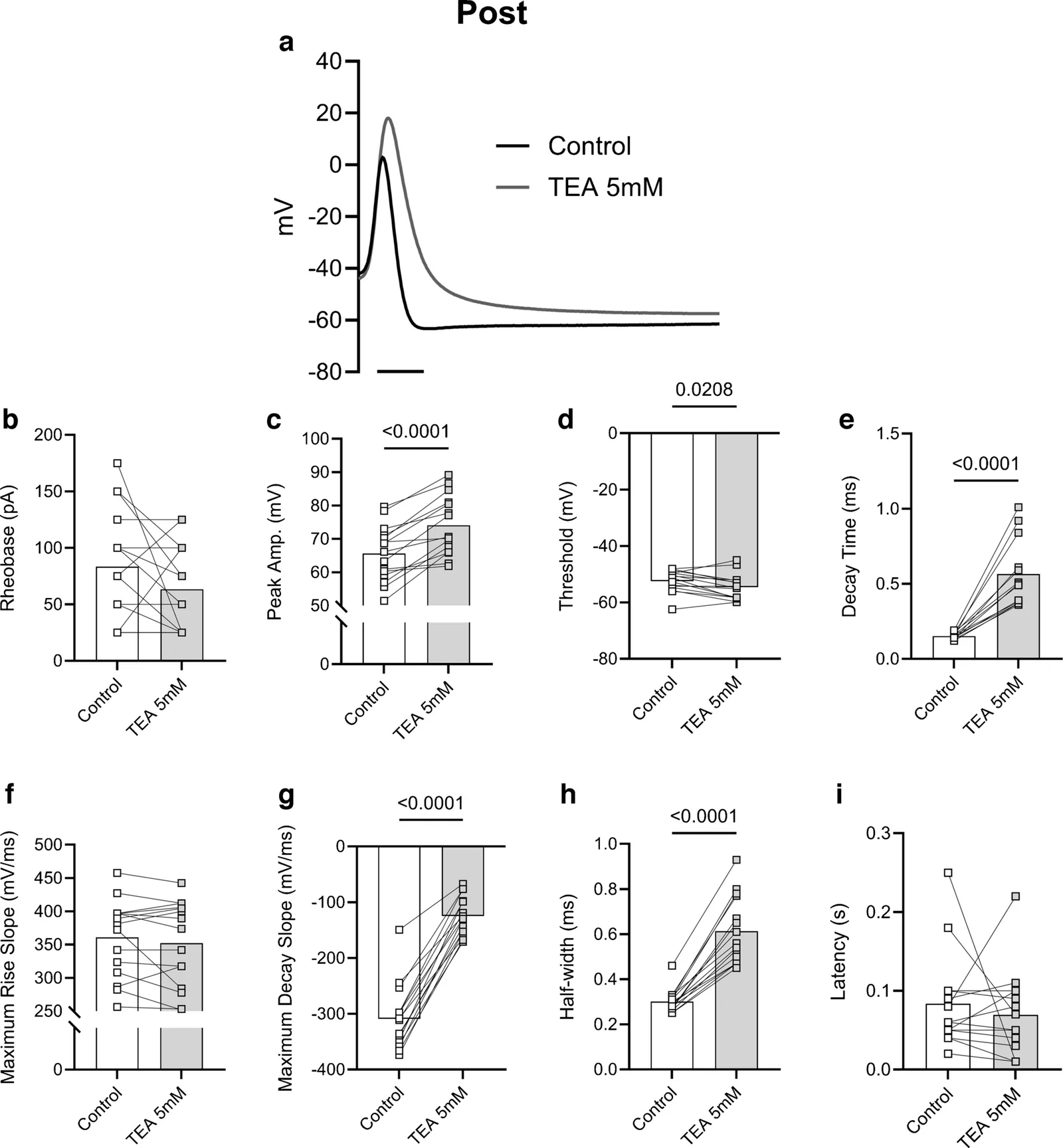
Effect of TEA on action potential parameters of post-hearing fusiform neurons, **a**. Representative example of a post-hearing action potential before and after TEA 5 mM; Time scale bar = 1 ms. **b**. Effect of TEA 5 mM on: **c**. Rheobase; **d**. Peak amplitude; **e**. Threshold; e. Decay time; **f**. Maximum rise slope. **g**. Maximum decay slope; **h**. Half-width; **i**. Latency

In fusiform neurons from post-hearing animals’ application of TEA 5 mM also changed several AP parameters (Fig. 5a). It did not affect rheobase (Fig. 5b), maximum rise slope (Fig. 5f) and latency (Fig. 5i). But it increased AP amplitude (from 65 ± 2 mV to 74 ± 2 mV; t = 6.727, df = 14, *p* < 0.0001, *n* = 15; Fig. 5c), decreased threshold (from −52. 4 mV to −54.6 mV; t = 2.603, df = 14, *p* = 0.02; *n* = 15; Fig. 5d), increased decay time (from 0.15 ± 0.006 to 0.56 ± 0.06 ms; t = 7.005, df = 12, *p* < 0.0001, *n* = 13; Fig. 5e), decreased maximum decay slope (from −309 ± 15 mV/ms to = 125 ± 9 mV/ms; t = 16.46, df = 14, *n* = 15; Fig. 5g) and increased half-width (from 0.3 ± 0.01 ms to 0.6 ± 0.4 ms; t = 10.49, df = 14, *p* < 0.0010, *n* = 15; Fig. 5h).

**Fig. 6:**
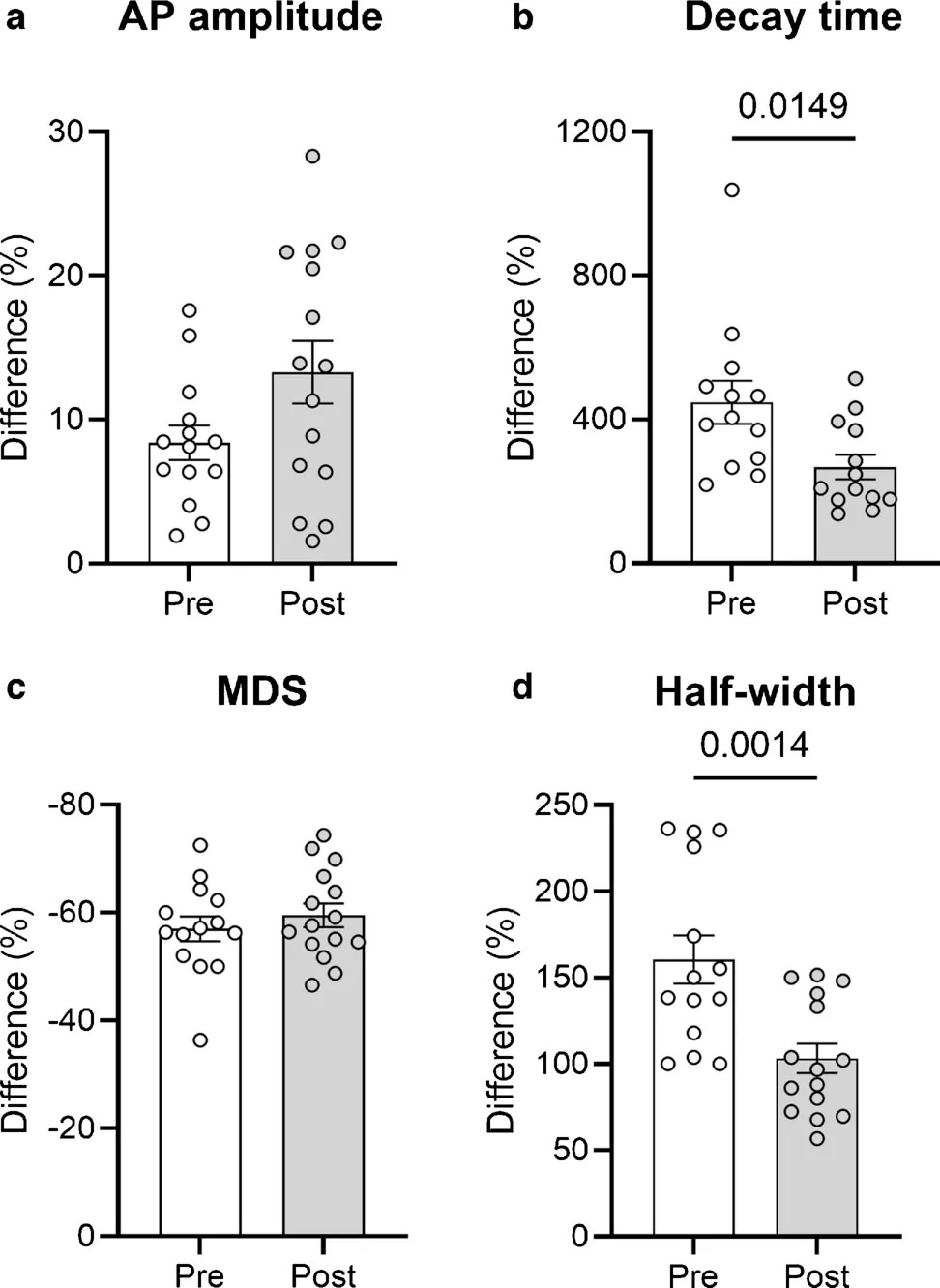
Effect of TEA on the: **a**. AP amplitude; **b**. Decay time; **c**. Maximum decay slope; **d**. Half-width

Interestingly, the effect of TEA was more pronounced in cells from pre-hearing animals. We compared the effect of TEA on 4 parameters that would be mostly affected by Kv3 channels (amplitude, decay time, maximum decay slope, and half-width). The effects on peak amplitude and maximum decay slope were similar in both cell groups (Fig. 6a,c), but we observed a more pronounced effect of TEA on decay time and half-width of action potentials in pre-hearing cells (Figs. 6b,d).

**Fig. 7:**
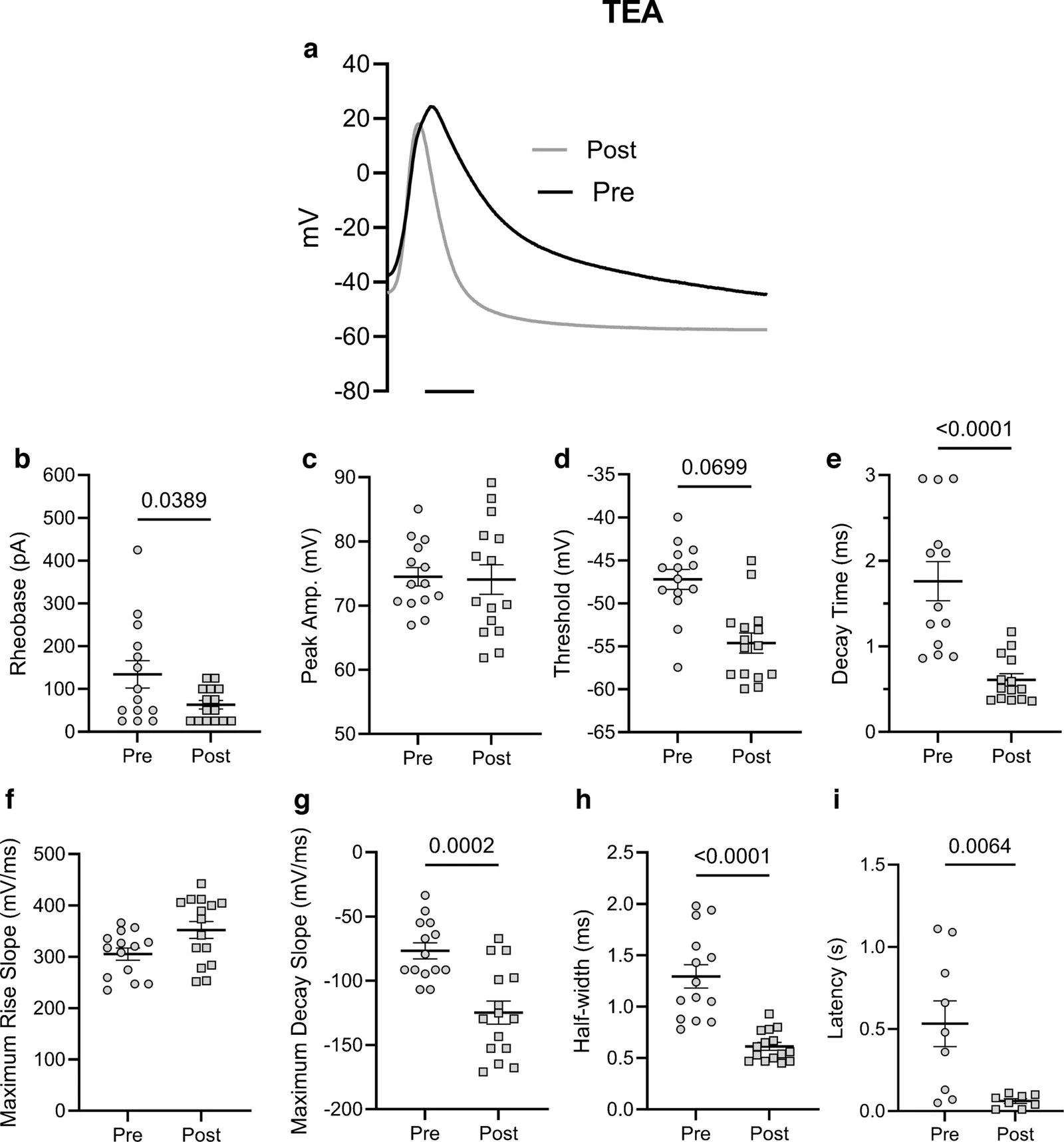
Comparison of action potentials after 5 mM TEA in pre-hearing and post-hearing neurons; Time scale bar = 1 ms. **a**. Representative example of a pre- and post-hearing action potential after TEA 5 mM. Comparison of pre- and post-hearing action potentials parameters after TEA 5 mM: **b**. Rheobase; **c**. Peak amplitude; **d**. Threshold; **e**. Decay time; **f**. Maximum rise slope. **g**. Maximum decay slope; **h**. Half-width; **i**. Latency

However, after TEA 5 mM, post-hearing fusiform neurons still present similar differences from pre-hearing neurons (Fig. 7a-i), suggesting that Kv3 channels are not responsible for the differences in AP waveform after hearing establishment.

### TEA Inhibits Action Potential Firing in Pre- and post-hearing Fusiform Neurons and Induces Burst Firing in Post-hearing Neurons

**Fig. 8:**
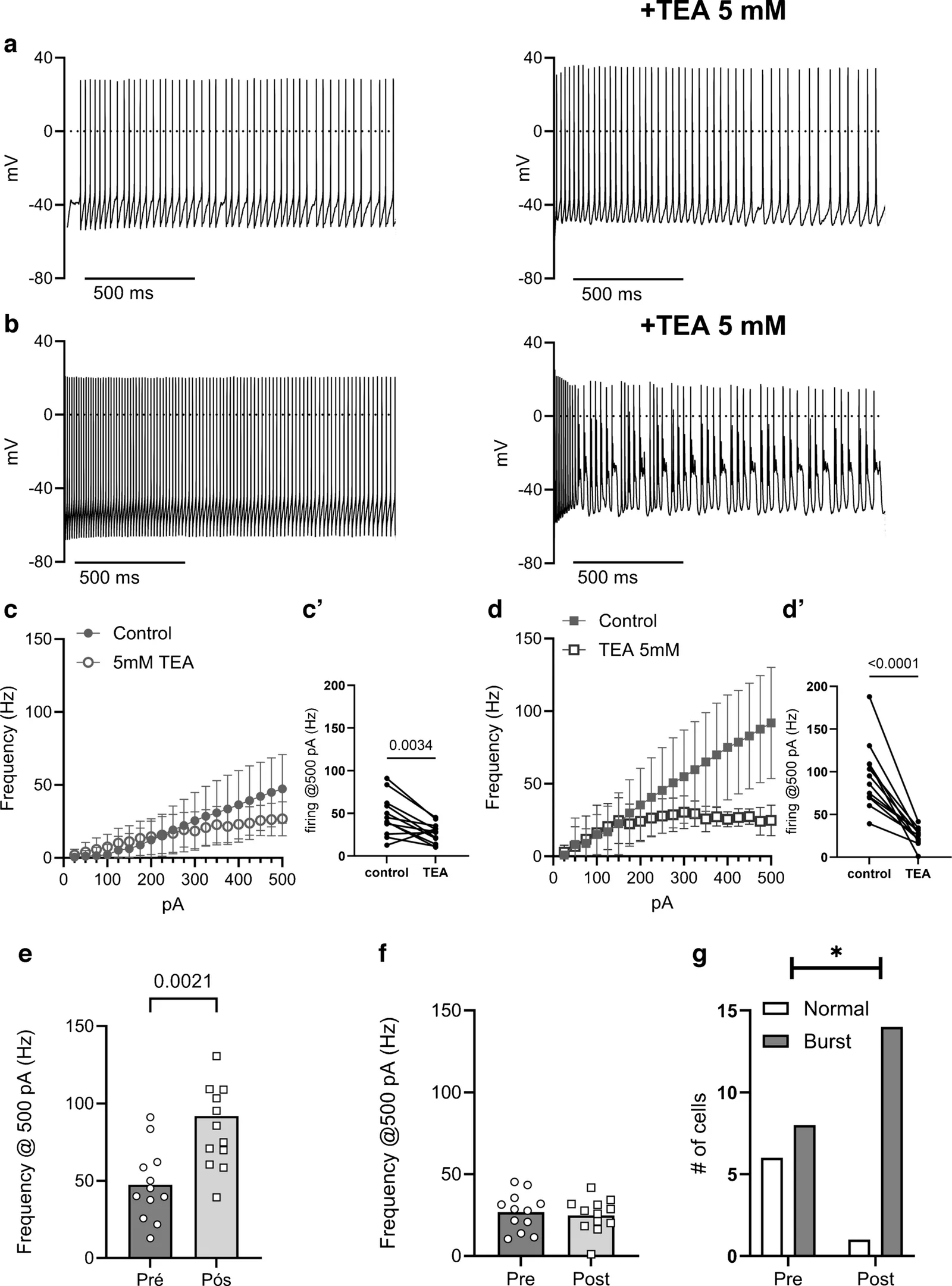
Effect of TEA on action potential firing. **a**. Representative example of the effect of TEA-5 mM on action potential firing (I = 225 pA) of a pre-hearing fusiform neuron. **b**. Representative example of the Effect of TEA-5 mM to action potential firing (I = 325 pA) of a post-hearing fusiform neuron. **c.** Frequency-intensity plot of action potential firing of pre-hearing fusiform neurons before and after TEA 5 mM. **c**’. Effect of TEA on firing in response to 500 pA current. **d**. Frequency-intensity plot of action potential firing of post-hearing fusiform neurons before and after TEA 5 mM. **d´.** Effect of TEA on firing in response to 500 pA current. **e**. Comparison of action potential firing (I = 500 pA) of pre-hearing and pos-hearing fusiform neurons. f. Comparison of action potential firing (I = 500 pA) after TEA 5 mM, of pre-hearing and post-hearing fusiform neurons. g. Comparison of TEA-induced burst firing in pre- and post-hearing neurons

We measured the impact of applying TEA on action potential firing. As previously reported [4], pre-hearing fusiform neurons have less ability to fire high-frequency action potential trains in response to progressive current injection than post-hearing neurons (Fig. 8a-d). Action potentials in response to 500 pA were smaller in pre-hearing neurons than in post-hearing neurons (pre-hearing: 47.3 ± 6.7 Hz., post-hearing: 91.9 ± 10.6 Hz. t = 3.468, df = 23, *p* = 0.002, unpaired t-test. n = 12 and 13 respectively; Fig. 8e). Application of TEA 5 mM significantly inhibited the firing in pre-hearing neurons (AP firing at 500 pA: control: 47.4 ± 7 Hz, TEA: 26.8 ± 3 Hz; t = 3.709, df = 11, *p* = 0.003, *n* = 12; Fig. 8c’). And in post-hearing neurons (AP firing at 500 pA: control: 94.7 ± 11 Hz, TEA: 24.8 ± 3 Hz; t = 6.695, df = 11, *p* < 0.0001, *n* = 12; Fig. 8d´). The effect of TEA was significantly stronger in post-hearing neurons (−69.8 ± 10 Hz.) than in pre-hearing neurons (−20.5 ± 5 Hz. t = 4,0.74, df = 22, *p* = 0.0004). After TEA 5 mM, the firing of both pre- and post-hearing neurons was similar (AP firing in response to 500 pA: pre-hearing: 26.8 ± 3 Hz., post-hearing: 24.8 ± 4 Hz., t = 0.438, df = 22, *p* = 0.66. *n* = 12 and 12 respectively; Fig. 8f).

Interestingly, after application of TEA 5 mM, several neurons displayed complex (burst) firing, similar to the ones observed in cartwheel neurons [39, 40], as observed by [27] (Fig. 8b). This behavior was more frequent in post-hearing neurons (14 out of 15 neurons, 93%) than in pre-hearing neurons in 8 out of 14 neurons (36%; p = 0.03, Fisher´s exact test; Fig. 8g).

### Burst Firing is Not Caused By Inhibition of Calcium-activated Potassium Channels

**Fig. 9:**
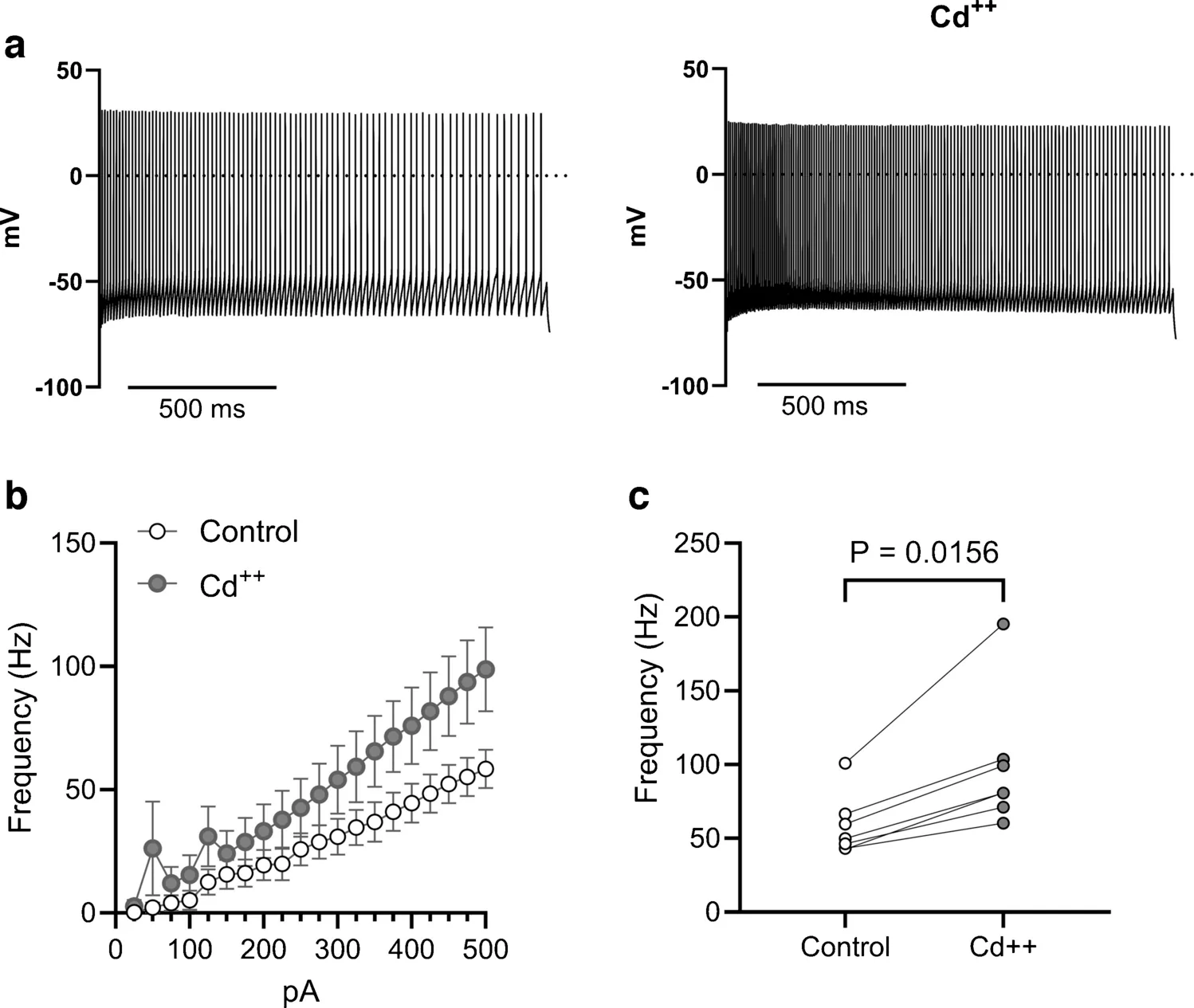
**a**. Representative action potential firing (I = 225 pA) before and after application of Cadmium Chloride 200 µM **b**. Frequency-intensity plot of action potential firing of post-hearing fusiform neurons before and after Cd^++^. **c**. Effect of Cd^++^ on firing in response to 500 pA current. All experiments were performed on post-hearing neurons

TEA at millimolar concentrations blocks other potassium channels, which should be considered when interpreting its effects [7, 41]. BK calcium-dependent potassium channels are sensitive to millimolar TEA, and their inhibition could lead to the burst firing observed after TEA in post-hearing neurons and by [27]. To determine whether inhibition of BK channels could account for the burst firing observed after TEA, we applied the voltage-gated calcium channel blocker Cadmium Chloride (200 µM) to block calcium influx during depolarizations, thereby preventing BK channel activation. Application of cadmium did not produce burst firing (Fig. 9a), and, unlike TEA, it increased AP firing (Fig. 9b,c). Cadmium did not significantly modify any AP parameters (Table 2).

**Table 2:** Comparison of action potential parameters from fusiform neurons from post-hearing neurons before and after cadmium chloride 200 µM

	Control	Cd^++^
Rheobase (pA)	114 ± 24 (*n* = 7)	82 ± 16 *p* = 0.078 (*n* = 7)
Peak amplitude (mV)	76 ± 1 (*n* = 7)	74 ± 1 *p* = 0.3 (*n* = 7)
Threshold (mV)	−45 ± 1 (*n* = 7)	−46.7 ± 1 *p* = 0.3 (*n* = 7)
Decay time (ms)	0.16 ± 0.01 (*n* = 7)	0.16 ± 0.01 *p* = 0.7 (*n* = 7)
Max. rise slope (mV.ms^−1^)	446 ± 9 (*n* = 7)	427 ± 15 *p* = 0.09 (*n* = 7)
Max. decay slope (mV.ms^−1^)	−352 ± 14 (*n* = 7)	−351 ± 17 *p* = 0.9 (*n* = 7)
Half-width (ms)	0.29 ± 0.008 (*n* = 7)	0.29 ± 0.01 *p* = 0.7(*n* = 7)
Latency (s)	0.096 ± 0.02 (*n* = 7)	0.093 ± 0.02 *p* = 0.9 (*n* = 7)

### TEA 1 mM Produced Smaller Effects than at 5 mM

**Fig. 10:**
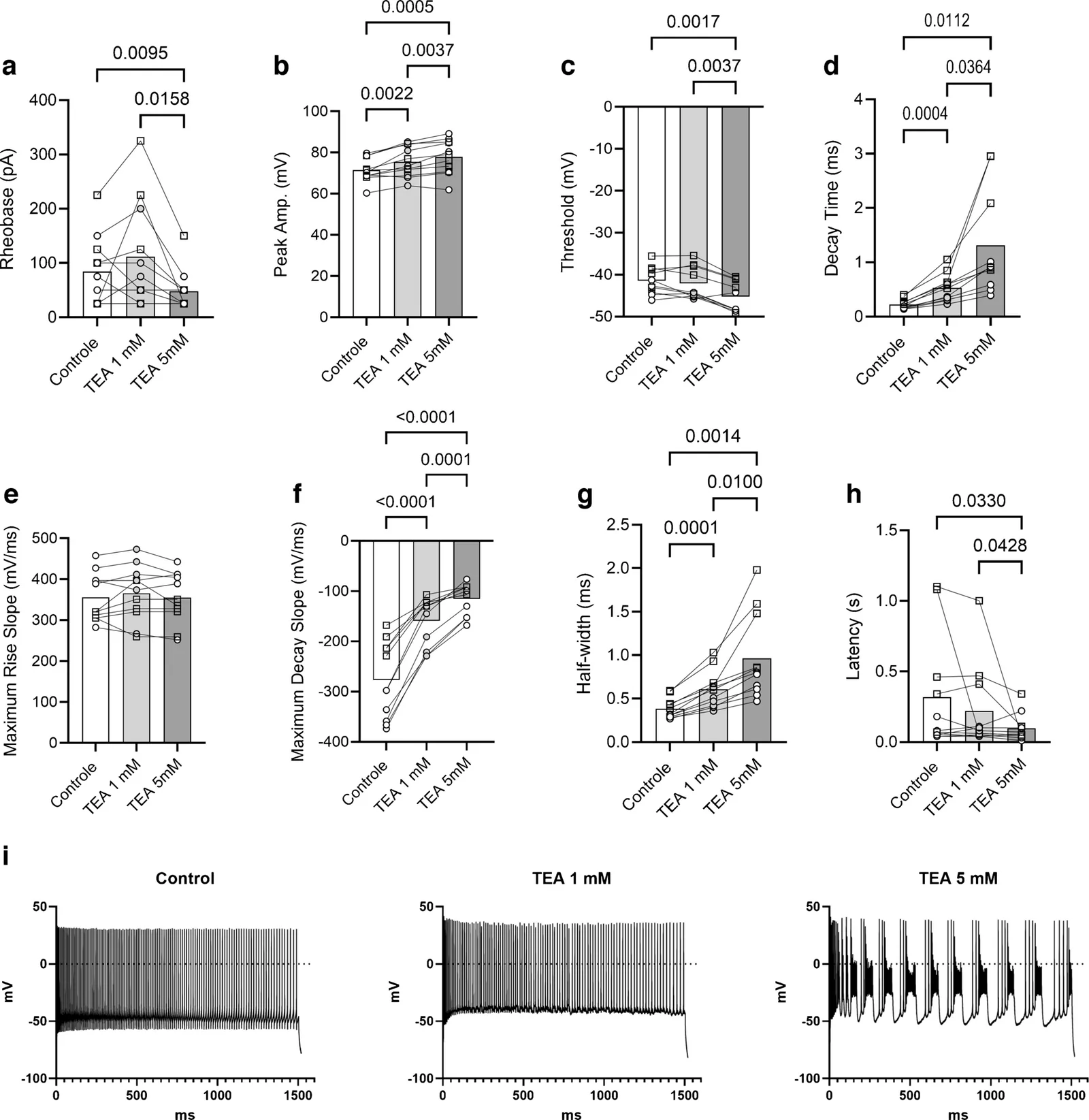
Comparison of action potentials after sequential application of 1 mM and 5 mM TEA in pre-hearing and post-hearing neurons **a**. Rheobase; **b**. Peak amplitude; **c**. Threshold; **d**. Decay time; **e**. Maximum rise slope. **f**. Maximum decay slope; **g**. Half-width; **h**. Latency. **i**. Action potential firing. Squares represent data from pre-hearing neurons (*n* = 5) and circles from post-hearing neurons (*n* = 6)

We also tested TEA at a concentration of 1 mM (followed by 5 mM), which is more often used to block Kv3 channels. We observed a smaller effect of 1 mM than of 5 mM TEA on increasing AP duration (Fig. 10a-h). Interestingly, 1 mM TEA did not induce burst firing, as observed in [27] (Fig. 10i). Moreover, the pre- and post-hearing AP differences remained even in 1 mM TEA (Table 3). On the other hand, TEA 1 mM did not affect AP rheobase, threshold, or latency, unlike TEA 5 mM, suggesting a more specific effect of TEA on Kv3 channels at 1 mM. Thus, although TEA 5 mM might block channels other than Kv3, we believe that under our experimental conditions, TEA 5 mM was more effective at completely blocking Kv3 currents than TEA 1 mM.

**Table 3:** Comparison of action potential parameters from fusiform neurons from pre-hearing and post-hearing neurons after TEA 1 mM

	Pre-hearing	Post-hearing
Rheobase (pA)	150.0 ± 56 (*n* = 5)	79 ± 27 *p* = 0.25 (*n* = 6)
Peak amplitude (mV)	74 ± 2 (*n* = 5)	75 ± 4 *p* = 0.8 (*n* = 6)
Threshold (mV)	−39 ± 1 (*n* = 5)	−45 ± 0.2 *p* = 0.006 (*n* = 6)
Decay time (ms)	0.7 ± 0.09 (*n* = 5)	0.36 ± 0.05 *p* = 0.006 (*n* = 6)
Max. rise slope (mV.ms^−1^)	331 ± 22 (*n* = 5)	394 ± 29 *p* = 0.13(*n* = 6)
Max. decay slope (mV.ms^−1^)	−120 ± 4 (*n* = 5)	−191 ± 18 *p* = 0.007 (*n* = 6)
Half-width (ms)	0.77 ± 0.08 (*n* = 5)	0.46 ± 0.04 *p* = 0.007 (*n* = 6)
Latency (s)	0.4 ± 0.2 (*n* = 5)	0.07 ± 0.01 *p* = 0.06 (*n* = 6)

## Discussion

Kv3 channels are linked to neurons' ability to fire high-frequency, tonic action potentials [42]. Fusiform neurons have the ability to fire high-frequency, regular trains of action potentials tonically in response to current injection [4, 26, 27, 31, 33] and Kv3 channels in fusiform neurons are fundamental to this ability [27]. Recent patch-seq analysis of the cochlear nucleus showed that Kv3.2 gene (Kcnc2) is associated with cells with tonic firing and short APs, as the fusiform neuron [43]. Thus, Kv3 channels are fundamental for the ability of the fusiform neuron to fire fast trains of short action potentials.

Kv3 channels play a key role in shaping various properties of action potentials (APs), mainly by shortening their duration [7, 27]. We found that TEA produced similar effects on the APs of fusiform neurons in both pre-hearing and post-hearing animals. TEA mainly increases the amplitude, half-width, decay time, and decay speed of the APs. These effects align with expectations from Kv3 channel inhibition. In contrast, parameters such as rheobase, latency, AP threshold, and maximum AP rise rate are minimally affected or unaffected by Kv3 blockade. These latter properties are primarily controlled by low-threshold potassium channels, like those in the Kv1 family [44, 45]. These effects on AP rheobase, threshold, and rising slope are probably caused by inhibition of Kv1 channels. Therefore, Kv3 blockade causes significant changes in AP half-width, decay time, and maximum decay rate, as seen in our results and as previously reported in the presynaptic terminal Calyx of Held [11]. Interestingly, the effect of TEA on half-width and decay time was substantially greater in pre-hearing neurons than in post-hearing neurons, indicating a differential impact of Kv3 channels inhibition on action potential waveform in pre- and post-hearing neurons.

APs of fusiform neurons from pre- and post-hearing periods differ significantly [4], and we hypothesize that increased Kv3 current expression may underlie these differences. Blocking Kv3 channels with TEA prolongs the APs from pre- and post-hearing fusiform neurons. Interestingly, this effect was more pronounced in pre-hearing neurons, suggesting that Kv3.1 channels have a stronger impact on their APs. However, the difference in AP parameters between these neurons persists after TEA, with post-hearing neurons remaining faster and having shorter APs than pre-hearing fusiform neurons. Therefore, although Kv3.1 channels are important to shorten the APs of both pre- and post-hearing neurons, the developmental differences observed in DCN fusiform neurons are then due to TEA-resistant Kv currents that are more prominent in post-hearing neurons or by developmental differences in voltage-activated sodium channels kinetics, as seen in the Calyx of Held synapse [46].

On the other hand, application of TEA (5 mM) inhibited the firing frequencies of both pre-and post-hearing fusiform neurons, but more strongly in post-hearing neurons, and increased burst firing in post-hearing neurons, suggesting that the increased expression of Kv3.1b channels in fusiform neurons is probably responsible for these cells' ability to fire short APs at higher frequencies.

The Kv3 family channels are characterized by an activation threshold at more depolarized membrane potentials (~ −10 mV) and are classified as high-threshold activated (HVA) potassium channels [10]. These channels are more sensitive to extracellular TEA than most Kv subunits, as they are blocked by 1–5 mM TEA [41]. We chose TEA 5 mM because in our preliminary experiments, TEA 1 mM did not induce burst firing as seen in [27]. We performed further experiments showing that 1 mM TEA had smaller effects on AP duration than 5 mM TEA, but did not alter the differences observed in the APs of pre- and post-hearing neurons, suggesting an incomplete block of Kv3.1 channels at this concentration. However, 1 mM TEA did not affect AP rheobase and threshold, suggesting that 5 mM TEA is affecting other Kv channels.

Kv1.1 subunits are sensitive to millimolar TEA concentrations and would also be inhibited by the concentrations of TEA we used. Kv1.1 channels are low-voltage-activated (LVA) channels, being activated around −40/−50 mV [44, 47]. However, the IV of the TEA-inhibited current is more typical of HVA Kv3 channels, with currents appearing around −20 mV (Fig. 1a'b') and half-activation voltages of −7 mV closer to the typical values of HVA currents. Therefore, we believe that the majority of the 5 mM TEA-sensitive current is attributable to HVA Kv3 channels, but the effects on AP threshold suggest that a fraction of the TEA-sensitive current is from Kv1 channels.

Calcium-activated potassium BK channels are blocked by low millimolar TEA [41, 48]. We applied cadmium chloride (200 µM) to block calcium entry through voltage-activated calcium channels and consequently to inhibit BK channel activation. Cadmium did not induce bursts, did not decrease firing, nor change AP waveform, but it did increase firing, which could also be caused by inhibition of SK channels. Thus, the effect of TEA was not due to inhibition of BK channels.

Kv2.2 and Kv7.2 channels are affected by low millimolar TEA, [41]. The Kv2.2 channel is a high-voltage-activated potassium channel with slower activation than Kv3 channels [47]. This channel affects the decaying phase of the action potential and the after-hyperpolarization, decreasing the refractory period by speeding the removal of sodium channel inactivation through a more pronounced hyperpolarization [49]. Interestingly, patch-seq experiments showed that the expression of the Kv2.2 channel is characteristic of DCN´s fusiform neurons, suggesting that a part of the TEA´s effect on action potential decay slope and firing frequency could be mediated by Kv2.2 channel inhibition, although their slow activation makes it less likely to affect the first action potential used in our analysis. However, Kv2.2 channels could be relevant to allow high-frequency firing in fusiform neurons, as seen in cartwheel neurons [50]. Kv7.2 channels are low-voltage-activated potassium channels with a very slow activation rate and are unlikely to affect the first action potential in a train used in our analysis; their inhibition would increase rather than decrease firing frequencies [51].

Our immunolabeling experiments showed a general increase in labeling intensity of the anti-Kv3.1b subunit antibody, reflecting increased expression of this subunit in all three layers of the DCN in post-hearing animals. This aligns with a study indicating that exposure to acoustic stimuli raises Kv3.1b expression in the rat auditory brainstem [52]. Interestingly, although fluorescence intensity increased in all 3 layers after P14, only in the fusiform cell layer was an increase in puncta number observed. The specific increase in puncta number in the fusiform cell layer might suggest an increased number of sites of Kv3.1b expression in the fusiform neurons after hearing. It is important to note that we cannot exclude that some of these puncta could also reflect pre-synaptic boutons.

It is possible that after hearing, we observe an increase in Kv3.1b expression at the expense of other Kv3 subunits. According to this hypothesis, the cerebellum exhibits a predominance of the Kv3.1a isoform until about P15, after which Kv3.1b becomes dominant [19]. This suggests that during development, a transition between subunit expression levels may occur, thereby maintaining the overall current despite the isoform switch. The Kv3.1b isoform has been shown to be more effective in supporting high-frequency firing than the Kv3.1a isoform [21] and it is associated with the axon initial segment, closer to voltage-gated sodium channels, thereby providing stronger control over action potential firing, whereas the shorter Kv3.1a isoform is more concentrated in the somato-dendritic compartment [20, 21].

We also tested whether hearing could alter Kv3.1b subunit phosphorylation after the onset of hearing at p14. Phosphorylation of the Kv3.1b channel decreases its ability to support high-intensity firing [37]. Noise exposure lowers Kv3.1b phosphorylation in the MNTB [38, 52]. However, we did not observe labeling for the phosphorylated form of Kv3.1b in the DCN of either pre-hearing or post-hearing animals. As a control for the antibody, we tested it in the MNTB, where phosphorylated Kv3.1b had previously been observed [38]. We observed labeling of the phosphorylated Kv3.1b isoform. Therefore, Kv3.1b is probably not phosphorylated in the DCN of mice during our subjects' age range and acoustic environment.

The impact of Kv3 expression on high-frequency firing is due to the high activation threshold of Kv3 channels, which open only during the AP, and their fast kinetics, which promote rapid repolarization, enabling neurons to fire APs at higher frequencies [47]. Additionally, Kv3.1b channels can produce a resurgent potassium current due to their quick activation and inactivation kinetics and the rapid relaxation of the voltage sensor [53]. This feature allows these channels to briefly reactivate during repolarization, thereby boosting the efficiency of subsequent spikes. Therefore, the increased expression of the Kv3.1b subunit after hearing enhances these properties of the Kv current, allowing post-hearing fusiform neurons to fire at higher frequencies than pre-hearing neurons. However, we cannot rule out the participation of Kv2 channels in this developmental difference at this point. Further studies with more specific pharmacological tools will be necessary to discern the role of Kv3 and Kv2 channels in allowing high-frequency firing in DCN fusiform neurons.

In conclusion, our results showed that DCN fusiform neurons in mice increase their Kv3.1b channel expression after hearing, possibly enabling them to fire high-frequency trains of fast action potentials. However, blocking Kv3 channels with TEA similarly affects Kv currents and the duration of APs in pre- and post-hearing neurons. Thus, increased Kv3.1b channel expression is not responsible for the differences in action potential kinetics observed after hearing. Because hearing deprivation and hearing onset do not affect the ability of these neurons to produce high-frequency firing [35], the observed changes in AP waveform, firing, and Kv3.1b expression are probably not dependent on acoustic stimulation after the opening of the ear canal at pP14.

## Data Availability

All data files are available on request from the authors.

## Declarations

### Artificial Intelligence Statement

We improved the writing using the Grammarly AI tool.
